# Observations upon the Importance and Value of a Knowledge of the Collateral Branches of Medicine and Surgery in Connection with Dentistry

**Published:** 1847-06

**Authors:** James Robinson

**Affiliations:** London.


### ARTICLE II.

***Observations upon the Importance and Value of a Knowledge of the Collateral Branches of Medicine and Surgery in connection with Dentistry.*** By JAMES ROBINSON, D. D. S., London.

To prove the importance and value of a general knowledge of medicine and surgery to the dental practitioner, we have only to refer to those cases, and they are by no means of unfrequent occurrence, in which an affection of the dental organs arises from, and is dependant on some gastric or constitutional irritation or derangement; for, although some diseases of the teeth are strictly idiopathic, arising in, and being confined to, those organs, there are others, obscure in their origin, intricate in their symptoms, and whose character, at different periods, is varied and uncertain.

The first description of these cases may, it is true, be successfully managed without any great amount of physiological or pathological knowledge; but in the second, the case is different, and unless the practitioner possess a general acquaintance, not only with these branches of science, but with medicine and surgery, he will be unable to combat the disease himself, and equally incompetent to decide when it may be necessary to call in the aid of a professor of medicine or surgery, to assist in its management.

To illustrate these opinions, we have only to name odontalgia, for instance, independent of its accession as an idiopathic disease, is by no means confined to local causes, but frequently resulting from a disordered state of the digestive organs, at others, from constitutional irritation, and, occasionally, as an accompaniment, if not a symptom, of pregnancy; while again, in some of the more complicated dental affections, it occasionally occurs, that cause and effect are so intimately blended by their action and reaction on each other, that an extensive knowledge of general physiology and pathology, combined with a discriminating judgment, are essential to a correct diagnosis, and no inconsiderate amount of medical and surgical knowledge necessary to their treatment.

The frequent occurrence of these cases in practice, must be more than sufficient to convince the most sceptical that the dentist should adopt an extensive range of study; that a *general* knowledge of every branch of science that can have relation to any one of these cases, is essentail to the accomplished dental practitioner, and that no person, however talented he may be in every other respect, can be said to be properly educated, the superstructure of whose knowledge has not been based upon an acquaintance with physiology and anatomy.

How is it possible that the dentist should be prepared to know the mal-position of parts, unless he be acquainted with their natural ones; how can he ascertain their morbid, and in some cases complicated action, unless he has a knowledge of their normal duties, and the laws by which they are governed? How can he combat disease, when he is ignorant of, and incompetent to investigate its cause?

It may be conceded, that in simple idiopathic diseases of the teeth, only a small amount of physiological and surgical knowledge is absolutely necessary; but even in these, the practice of the educated and scientific dentist will be the most decisive, satisfactory and beneficial.

Anatomy teaches the structure and position of the different parts of the body; physiology, their uses and employment. Now, while it might be assumed, *prima facia*, that it would be of no advantage to the practitioner, whose duties, strictly speaking, are confined to the head and face, to study all the various complications of general anatomy; while it may be thought that the structure of the foot or the mechanism of the wrist have little to do with diseases of the teeth and mouth, we find such, however, is not the case, and we fearlessly assert that it is impossible to perfectly understand the one, without a general acquaintance with the other.

The importance of anatomy to the surgeon, is admitted by all; what would be thought of that man, who, because he did not intend to practise as a dentist or oculist, omitted the mouth and the eye from the range of his studies; and yet, too frequently, a similar plan is adopted by the dentist, who confines his studies *entirely* to the teeth. The science of anatomy must be studied as a whole, neither is or can there be a line of demarcation at which the anatomy of the dentist terminates. The nerves and blood-vessels that supply the teeth and mouth are derived originally from the same source, subject to the same laws, and have the same intercommunications and sympathies as those that supply the other parts of the body, and these can only be properly understood by a general knowledge of the whole.

The anatomy of the head and face must unquestionably form the leading and paramount study of the dentist, as it is to these parts his professional duties are more particularly addressed and which are composed of a variety of bones minutely supplied with nerves and blood-vessels, and possessing some of the most intricate muscular arrangements in the body, these parts afford an interesting field for study and observation, but the studies of the dentist must not end here, for such is the sympathetic communication between these parts and the rest of the body, that cases of odontalgia frequently occur from the application of cold to the lower, or some specific irritation of the upper extremities, cases in which relief is not to be obtained by reuniting directly to the parts affected, but by general or constitutional treatment. We know, also, that in these and similar cases, tooth after tooth has been extracted in the abortive attempt to afford relief, while from the want of general knowledge on the part of the practitioner, the real cause of the disease and legitimate means of cure have been completely overlooked.

Again, there are other cases in which a knowledge of general anatomy becomes more decidedly evident in which disease of the teeth may be traced to some functional derangement, and in which, unless the practitioner has an acquaintance with general physiology, he must be perfectly helpless.

Nor must we forget that while the morbid state of other parts may give rise to diseases of the teeth—diseases of these organs have a reciprocal action producing affections of the eye, the ear, and the stomach, the rationale of which may be traced by a knowledge of the intercommunication of the nerves of the respective parts, but must be inexplicable to those unacquainted with their structure.

Aware of their parts, how ridiculous would a dental practitioner appear, in replying to a series of questions relative to the above subject, if he were to say, "Oh my knowledge is confined only to the *teeth*, the nerves of which possibly may communicate with those of other parts, but of the nature and extent of their communication I am ignorant;" and yet were some practitioners to exercise as much candor as they do quackery, such a confession would be of no unfrequent occurrence.

As then it is impossible to define where the line of demarcation terminates, or how far, or in what cases an extensive knowledge of anatomy may come into request to the advantage of the patient, and the reputation of the practitioner—the dentist who proposes to practice, scientifically, should not be content with a mediocre knowledge of the subject, but study anatomy, physiology and pathology as the proud work of his profession. Keeping in mind that it is impossible to separate these studies into parts, as reasonably might a person expect to understand the geographical position of England, while ignorant of the relative position of other countries, as a dentist to make any advantageous use of an isolated portion of anatomy and physiology. These studies, then, must be considered as a whole and in connection with each other, and inseparable, and as such constitutes the foundation on which the particular professional superstructure must henceforth be built.

For, however accurate our acquaintance with the parts of which the human body is composed, it would be of comparatively little value if unaccompanied by a knowledge of their uses and employments, so that anatomy may be considered as the handmaid of physiology which gives this information, whilst physiology may be described as teaching the uses of the different parts of the human body in their normal state.

It is asserted by some, of not very liberal or comprehensive minds, that a knowledge of general anatomy and physiology is unnecessary to the dental practitioner. We would ask, do not the teeth form a part of the animal economy, are they not like other parts formed from the general system, are they not supplied with blood-vessels and nerves from the same general sources, are they not acted upon by constitutional weakness or derangement, impaired by the influences of general ill health, and in some instances destroyed by the effects of general disease? A course of mercury carried beyond a certain extent, loosens and destroys the teeth, though it does not come into absolute contact with them, but is carried into the system by means of the absorbent glands, some putrid diseases act in the same manner by vitiating the fluids of the mouth; indeed, most complaints, it is well known, have more or less influence on these organs. How is the first process of their formation, their shedding and reproduction to be understood excepting by the aid of physiology? Did the teeth form an isolated portion of the human economy, were they not dependant on the great reservoir for their supply of blood, were their nerves not derived from the same universal source as those of other parts of the body, one might require the duty of the dentist to begin and end with the teeth; but while, on the contrary, they are subject to the same general influences of health and disease, dental surgery imperatively demands an intimate acquaintance with those general laws by which the animal economy is regulated.

The natural step from physiology, or the normal duties of the different parts, is to pathology, which treats of their diseased actions and changes, and as it is impossible to understand the latter, without an acquaintance with the former, so would that be of little avail unless supported by its fellow science, for how could we possibly distinguish the morbid action of parts, if we are unacquainted with their healthy duties. How separate sympathetic, or idiopathic complaints, from organic or structural disease, unless by an intimate acquaintance with the symptoms and appearances that mark the influence.

Thus, then, while by the aid of anatomical knowledge, we at once ascertain any mal-position of parts, while pathology points out the changes and ravages caused by disease, still, unless a knowledge of physiology be combined so as to understand not only the natural functions and duties of the different parts but the various processes by which they are originally found and by which nature assists in refusing any derangement from their normal state, the dentist will practice in the dark, his means of diagnosis and of cure will be equally limited and unscientific, instead of being able to assist nature and take advantage of her efforts, he will be in constant danger of contravening them, the most simple cases may be converted into serious ones, and the results of his practice will be as unsatisfactory as the practice itself is deficient in reason and science.

It might be thought, a priori, that a knowledge of those parts to which the practice of a dentist is restricted, is all that is necessary, and were these parts free from constitutional influence, were they independent of the other parts of the body, such might be the case, but the reverse of this is the fact, the formation of a tooth or the reproduction of any of the soft parts of the mouth requires the whole machinery of the human economy.

It is true that the medical and, strictly speaking, surgical knowledge of the dentist may not frequently be required, that he may not in many cases be called upon to exercise a general plan of treatment, as custom transfers the cases in which this is required (at least so far as the general treatment and the more formidable operations on the jaws are concerned) to the physician and operating surgeon, still, however, he must possess a certain amount of general knowledge and information, or he will be unable to distinguish the cases that legitimately come under his care, from those in which it is customary to call in the aid of a practitioner of medicine or surgery.

